# Therapeutic Drug Monitoring in Psychiatry: Enhancing Treatment Precision and Patient Outcomes

**DOI:** 10.3390/ph17050642

**Published:** 2024-05-16

**Authors:** Letizia Biso, Stefano Aringhieri, Marco Carli, Marco Scarselli, Biancamaria Longoni

**Affiliations:** 1 Department of Translational Research and New Technologies in Medicine and Surgery, University of Pisa, 56126 Pisa, Italy; l.biso@studenti.unipi.it (L.B.); carlimarco@outlook.it (M.C.); marco.scarselli@unipi.it (M.S.); 2 Mental Health and Pathological Addiction Department, AUSL Romagna Forlì-Cesena, 47121 Forlì, Italy; stefano.aringhieri@auslromagna.it

**Keywords:** therapeutic drug monitoring, mood stabilizers, psychiatric disorders

## Abstract

Psychiatric disorders often require pharmacological interventions to alleviate symptoms and improve quality of life. However, achieving an optimal therapeutic outcome is challenging due to several factors, including variability in the individual response, inter-individual differences in drug metabolism, and drug interactions in polytherapy. Therapeutic drug monitoring (TDM), by measuring drug concentrations in biological samples, represents a valuable tool to address these challenges, by tailoring medication regimens to each individual. This review analyzes the current landscape of TDM in psychiatric practice, highlighting its significance in optimizing drug dosages, minimizing adverse effects, and improving therapeutic efficacy. The metabolism of psychiatric medications (i.e., mood stabilizers, antipsychotics, antidepressants) often exhibits significant inter-patient variability. TDM can help address this variability by enhancing treatment personalization, facilitating early suboptimal- or toxic-level detection, and allowing for timely interventions to prevent treatment failure or adverse effects. Furthermore, this review briefly discusses technological advancements and analytical methods supporting the implementation of TDM in psychiatric settings. These innovations enable quick and cost-effective drug concentration measurements, fostering the widespread adoption of TDM as a routine practice in psychiatric care. In conclusion, the integration of TDM in psychiatry can improve treatment outcomes by individualizing medication regimens within the so-called precision medicine.

## 1. Introduction

**Figure 1:**
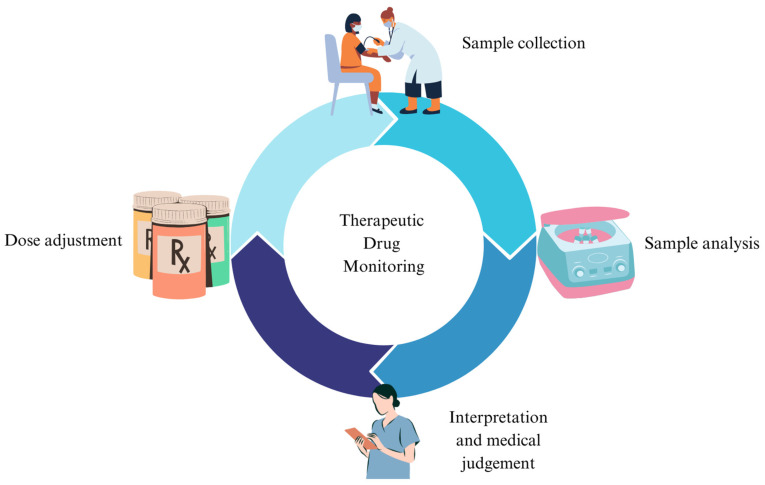
Schematization of the therapeutic drug monitoring (TDM) process.

Therapeutic drug monitoring (TDM) quantifies medications in biological tissues (usually in plasma or serum), generally under steady-state conditions [1]. The scope of TDM is to increase the safety and management of different drugs and to aid clinicians in decision-making regarding tailored therapy (Figure 1) [2,3]. 

In the past decades, a task force of the Arbeitsgemeinschaft für Neuropsychopharmakologie und Pharmakopsychiatrie (AGNP) was created to define the TDM consensus guidelines, which were initially published in 2004 [4] and then updated in 2018 [5]. The AGNP defines four levels of recommendation for performing TDM in different drug classes, depending on the existing evidence in support of drug monitoring: level 1, or “strongly recommended”, level 2, or “recommended”, level 3, or “useful”, and level 4, or “probably useful”. Level 1 includes drugs with well-established therapeutic reference ranges, for which TDM represents a useful tool in terms of dose titration, regular monitoring, and safety. Level 2 includes drugs with therapeutic ranges that have been acquired from drug concentrations at effective doses, and in which the use of TDM has an advantage, mainly in dose titration or problem solving. Level 3 includes drugs in which TDM can be used for special indications or problem solving, while level 4 refers to drugs for which the benefits of routinary TDM have not been established, but for which TDM could be potentially useful in particular cases [1].

A recent systematic review by Yi and colleagues evaluated the quality of more than 90 guidelines for TDM based on the Appraisal of Guidelines for Research and Evaluation (AGREE) II Instrument [6,7]. Considering the TDM guidelines for Central Nervous System (CNS) drugs, the overall quality of Hiemke and colleagues’ work was among the highest, with a score of 66.67%. The AGNP TDM task force also implemented another guideline, specifically on antipsychotic TDM, which scored 72.22% overall in the same systematic review and was therefore recommended for its usefulness [8].

**Figure 2:**
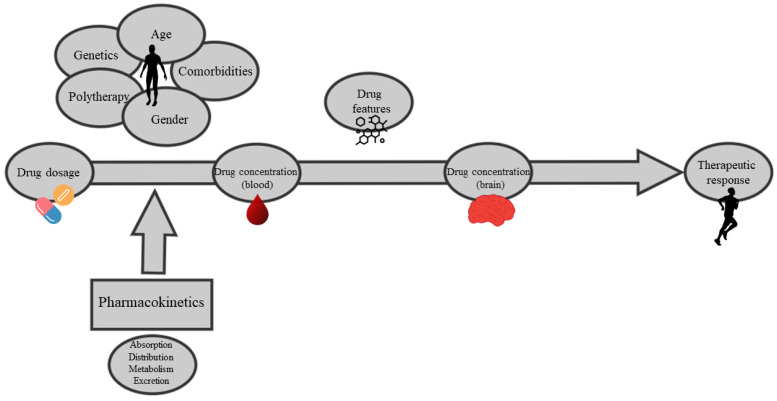
The role of therapeutic drug monitoring (TDM) in the process that leads to pharmacological response.

In the field of neuropsychopharmacology, TDM in mental health treatments represents an important topic within the scope of treatment individualization, minimizing adverse drug reactions (ADRs) and maximizing drug efficacy [5]. Moreover, treatment adherence can be monitored, thus reinforcing patient–clinician dialogue in a shared decision-making approach. The role of TDM is of particular importance when considering pharmacokinetic variables (Figure 2). The importance of TDM in patients with psychiatric disorders has been underlined for decades, with different studies reporting not only its efficacy for specific medications, but also its cost-effectiveness [9,10,11]. In the real-world clinical practice, however, the implementation of regular TDM is not always possible. For example, research conducted by Al Mutarid and colleagues found that although the practice of TDM was well known among doctors in Saudi Arabia, its actual use was poorly applicated, especially in smaller hospitals, mainly because of lack of resources [12]. Another study, conducted in Turkey by Eryılmaz and colleagues, found a positive approach in the frequency of TDM use among Turkish clinicians for mood stabilizers, with 98.4% of clinicians declaring regular use of TDM, especially for lithium and valproate, but with significantly lower rates of monitoring for other classes of psychotropic medications [13]. A survey conducted in China among psychiatric facilities by Guo and colleagues found that even when TDM was used to monitor the patients’ drug levels, in most cases, this was not followed with recommendations on dose adjustment, underlining the fact that sometimes there could be a lack of communication between clinicians and laboratory professionals [14]. These findings show that there is still room for improvement in the implementation of TDM in clinical practice in different settings around the world, and that there is a compelling need for information about correct TDM practices among health-care professionals. Drug monitoring also represents a way to minimize adverse events in patients taking psychiatric medications because it helps the clinician during the titration phase and follow-up, especially with drugs such as clozapine, which can be associated with potentially serious adverse events [15,16].

Appropriate use of TDM in this particular population is also of major importance because, in numerous circumstances, a combination therapy of multiple drugs is needed to achieve clinical stability, so both efficacy and safety must be thoughtfully considered, as well as drug–drug interactions [17,18]. Lastly, although these issues are often underestimated and possibly underdiagnosed, the interactions between psychiatric drugs and other substances, such as alcohol, tobacco, or other compounds, should be taken into account in order to monitor (and possibly predict) correctly changes in plasmatic drug levels [19,20].

The aim of this narrative review is to analyze the state of the art of TDM among medications used in psychiatric disorders. We will discuss the role and evidence of TDM for each medication under examination, based on the most recent literature. We will also analyze if and how TDM is implemented in the real-world clinical practice, based on the existing literature. Lastly, we will also briefly mention the novel and less-invasive monitoring approaches that could facilitate TDM in the future for both clinicians and patients.

## 2. TDM and Mood Stabilizers

In mood disorders, such as bipolar disorders, the most used medications backed by the current guidelines are lithium, valproate, lamotrigine, carbamazepine, and oxcarbazepine [21,22]. Table 1 shows the main mood stabilizers, with their therapeutic ranges, AGNP recommendation levels, and their principal sampling methods, including the experimental ones.

The history of lithium therapy goes back in time to the 19th century, even though introducing lithium for the treatment of manic episodes is credited to John Cade in 1949 [38]. To this day, lithium remains the most effective therapy for maintenance treatment and relapse prevention in bipolar disorder, but it can also be used in recurrent depression [39]. The downside of lithium treatment is a low therapeutic index (approximately 2); in other words, lithium has a low ratio between the dose (and therefore its serum levels) associated with toxicity and the dose that promotes a beneficial effect [40]. Lithium toxicity includes a wide variety of presentations, such as renal, cardiac, neurological, and endocrine disorders; at the same time, a suboptimal treatment with lithium because of excessively low dosages, scarce adherence, or abrupt discontinuation can lead to relapse [41].

This limitation leads to the necessity of keeping serum levels in a strict range, hence the importance of TDM. A systematic review of optimal lithium serum levels found that, based on current research, serum levels of adult patients should remain between 0.6 and 0.8 mmol/L, but they could be lowered to 0.4–0.6 mmol/L in case of sufficient response and poor tolerance, and they could be at the higher range of 0.8–1.0 mmol/L in case of suitable tolerance but suboptimal response [42]. The AGNP consensus guidelines accept a therapeutic range between 0.5 and 1.2 mmol/L but suggest that higher plasmatic levels should be used in the treatment of the acute phases, while in a maintenance treatment setting, it is advised to keep lithium plasma levels in the 0.5–0.8 mmol/L range. TDM is mandatory for lithium for safety reasons, and it is nowadays considered a standard of care, with a level 1 recommendation [5]. 

Considering lithium half-life (14–30 h), steady-state concentrations are reached in about 5–7 days from the beginning of treatment, and blood sampling should be performed after 12 + 1 h from the last administration (usually the evening dose) [42]. A cross-sectional study suggested that a 24 h sampling after the last dose may be more accurate for once-daily formulations to avoid overestimating plasmatic levels [43]. According to Mahli and colleagues, lithium monitoring should be performed after the first two days of treatment, then on the 7th day, at two weeks, after a month, at three and six months, after one year, and then at least yearly, at every dosage change, or whenever there is suspicion of toxicity, relapse, or lack of adherence. Cardiac, renal, and thyroid functions should be monitored as well in order to prevent organ damage [44]. The National Institute for Health and Care Excellence guidelines, on the contrary, suggest that lithium should be monitored every 3 months, with the organ function parameters to be monitored twice a year [45], while the British Association for Psychopharmacology guidelines suggest that lithium should be tested every 3–6 months [46]. 

Despite the importance of lithium monitoring, the frequent assessments can be perceived as inconvenient by patients, and clinicians may be discouraged from prescribing this life-saving medication [47,48]. In recent years, alternatives to blood testing, such as saliva sampling and machine learning approaches, have been proposed to facilitate lithium adherence and acceptance. Specifically, a recent study by Parkin and colleagues evidenced a good correlation between saliva and blood samples in patients taking lithium, opening the possibility for easier monitoring, which could be performed at the patients’ home [23]. Other biological fluids that are being tested for possible future applications in routinary lithium sampling include urine, sweat, interstitial fluid, and dried blood or plasma spots [24].

Another study by Hsu and colleagues showed that the use of machine learning algorithms such as the Support Vector Machine (SVM) could predict lithium concentrations in patients, and these models could soon be implemented in clinical practice, potentially in order to reduce the need to take samples from the patients’ blood [49].

Different studies have shown that lithium monitoring is often underused, despite the existing recommendations [50,51], even though, in some instances, the importance of regular TDM has been acknowledged by clinicians, with promising results in terms of ADR prevalence [52]. In other cases, while TDM seems correctly used in terms of frequency, it does not induce the clinician to adjust lithium dosages in the case of suboptimal plasma levels, possibly due to concerns about ADRs [45,53,54,55].

Valproate has also been used for decades, having been first introduced in the 1960s for the treatment of epilepsy and later for the treatment of manic episodes in bipolar disorder [56]. The level of recommendation for valproate TDM is 1, and the valproate therapeutic range is usually between 50 and 100 μg/mL. However, in patients with acute mania, higher plasma concentrations (up to 120 μg/mL) are acceptable if tolerated [5]. It is crucial to regularly monitor valproate, especially if the patient is taking other medications, because valproate is a cytochrome p450 inhibitor and can increase the concentration and, therefore, the effects of other concomitant drugs, possibly increasing the incidence of ADRs [57].

Valproate ADRs include neurological, gastrointestinal, hepatological, and hematological symptoms, which are usually more common in the first phases of treatment. It is the first cause of drug-induced hyperammonemia, and idiosyncratic hepatotoxicity and pancreatitis have been described [58,59]. Valproate should be avoided during pregnancy, and its use in women with childbearing potential should be carefully evaluated (and, if feasible, alternative drugs should be considered) because of its increased teratogenicity [60]. Valproate has been associated with increased risks of neural tube defects, cognitive impairment, craniofacial and orofacial cleft, cardiac malformations, and skeletal and limb defects [61].

Before initiating valproate therapy, liver function, ammonia levels, the body mass index (BMI), coagulation, whole blood and platelet count, and pregnancy should be assessed. Liver function, blood count, and the BMI should be assessed at three and six months, after one year of treatment, and at least annually after that. Valproate plasma concentration should be monitored every three to six months, and whenever a change in dosage is needed or other conditions occur [21,46]. The presence of hypoalbuminemia should also be monitored because, in this case, valproate levels may be inaccurate, considering that the albumin binding rate can be up to 90% [62]. The appropriate time to draw blood samples is 12 h after the last administration for regular valproate and 24 h for once-daily formulations [21,63]. In order to facilitate regular monitoring, alternatives to blood sampling are in the process of being implemented, such as saliva sampling and machine learning algorithms, which have the potential to reduce the required number and frequency of samplings. In particular, in 2015, Dwivedi and colleagues demonstrated a good correlation between saliva and serum samples in valproate monitoring. The study included patients with epilepsy and was conducted with the intention of helping to establish less-invasive means of TDM [25]. Future research is needed to further confirm these findings and to expand this correlation to patients with other disorders, such as affective disorders. Other researchers have found good reliability in dried blood spots and urine monitoring in patients with epilepsy [26,27]. Another way to reduce the need for frequent valproate monitoring is the use of predictive models, such as linear regression, logistic regression, SVM, random forest, and extreme gradient boosting (XGBoost), as successfully demonstrated by Hsu and colleagues in a recent paper [64].

Despite its proven benefits, valproate TDM is still an underused or misused tool for the clinician: some studies show low frequency of monitoring, while others show that valproate levels are often found underdosed or at the lowest-end limits in patients with mood disorders, which could either indicate a low adherence to therapy, or the use of low dosages by clinicians for the treatment of psychiatric disorders [55,65,66,67,68].

Carbamazepine is an effective drug for the treatment of acute mania, bipolar depression, and maintenance treatment in bipolar disorder. The safety risks and the general low tolerability of this drug, however, have made carbamazepine a second-line treatment (and third-line treatment for bipolar depression) for mood disorders [21]. 

As with lithium, TDM for carbamazepine is mandatory for safety reasons (level of recommendation: 1), and it is considered standard of care [5]. Carbamazepine ADRs can include vision problems, hyponatremia, confusion, urinary retention, and agranulocytosis. Hyponatremia and agranulocytosis, in particular, can be life-threatening conditions, although uncommon [69]. Another potential long-term effect of carbamazepine is decreased bone density, which may lead to osteoporosis [70]. The use of carbamazepine during pregnancy has been associated with an increased risk of spina bifida in the newborn, although it seems that the risk is milder compared to valproate [71]. More recently, carbamazepine during pregnancy has been associated with poorer academic performances in teenagers who had been exposed to the drug in utero compared to unexposed peers [72]. These concerns show the importance of scrupulous monitoring of this drug.

The accepted therapeutic drug range of carbamazepine is 4–12 μg/mL, and the compound bounds strongly to plasma proteins. The pharmacokinetic of carbamazepine is non-linear, as it auto-induces itself. It is important to remember that carbamazepine-10,11-epoxide, its metabolite, is an active compound which contributes to the drug effects, and that it can be increased when other anticonvulsant drugs, such as valproate, are used in concomitance [5,73]. Although the monitoring of the active metabolite itself does not seem necessary in monotherapy, it should be considered in patients taking other drugs that could have a pharmacokinetic interaction with carbamazepine to reduce the risk of ADRs [74].

When initiating carbamazepine therapy, patients should be instructed about the risk for skin rashes and the rare Stevens–Johnson syndrome, which are more common during the first weeks [21]. The risk for Stevens–Johnson syndrome seems higher in the population with the HLA-B*1502 allele [75]. Sodium levels should be checked at least annually, while blood pressure, lipid profile, and fasting glucose should be assessed at three and six months from the start of the treatment, and then annually. The BMI should also be checked, especially if the patient is on concomitant atypical antipsychotic therapy. Blood sampling for carbamazepine monitoring should be taken 12 h after the last dose, every six to twelve months [21]. Saliva sampling has also been considered as a valid alternative to standard blood sampling in carbamazepine TDM [28]. A study conducted in Indian patients with epilepsy revealed a good correlation between saliva and serum sampling, although the significance was lost in the case of polytherapy with more than three antiepileptic medications [29]. The validity of this less-invasive method was also more recently confirmed by another group of researchers, who demonstrated a good repeatability of saliva sampling [30]. Other studies have shown the possibility to measure carbamazepine levels in urine samples with good accuracy [31,32]. As for other drugs, the TDM of carbamazepine (and often the other required screening tests) is not performed enough, especially in mental-health settings [76,77], despite being a mandatory assessment.

The role of oxcarbazepine in the treatment of mood disorders is less documented. Its use was studied mainly in the 1980s for the treatment of manic episodes and was found to be comparable to other medications. It could be used in patients who do not tolerate carbamazepine [69]. The role of oxcarbazepine in treating depressive episodes and in maintenance is still unclear [78]. It has been proposed as an effective adjunctive treatment for lithium [79,80]. The ADRs of oxcarbazepine are pretty similar to carbamazepine; there seems to be a comparable risk for hyponatremia, as well as the risk of Stevens–Johnson syndrome, which is mainly present in specific Asian populations [69]. Teratogenicity seems lower compared to carbamazepine, and it is not dissimilar to that of the general population [81]. Because of its lower ability to induce CYP3A4 and to inhibit CYP2C19, compared to carbamazepine, oxcarbazepine does not induce itself, and it has fewer interactions with other drugs; this metabolic difference could make oxcarbazepine more tolerable than carbamazepine [82]. The oxcarbazepine plasmatic therapeutic range is 10–35 μg/mL, and it is usually measured with 10-hydroxycarbazepine, which is the active compound in vivo; the oxcarbazepine monitoring recommendation level is 2 [5]. Other researchers have proposed urine sampling as a reliable and alternative method to plasma sampling [33]. The necessity for routine TDM is still not entirely clear. However, it is beneficial in specific situations [83]. It is important to note that the majority of research regarding oxcarbazepine TDM is conducted on epilepsy treatment rather than psychiatric disorders.

Lamotrigine is an antiseizure drug approved for the treatment of bipolar disorder in monotherapy or adjunctive maintenance therapy, especially for the treatment and prevention of depressive episodes [84]. Lamotrigine, on the contrary, does not seem valid for the treatment of manic episodes, and it is therefore not recommended [21]. Relatively common ADRs are dizziness, nausea, and vomiting. It can cause mild-to-severe skin rashes, more commonly when co-administered with other medications, such as valproate, and less likely to occur with a slow titration (limiting its use in acute settings) [85]. The risk for Stevens–Johnson syndrome in patients taking lamotrigine has to be mentioned even if it is relatively low, often associated with specific HLA alleles (particularly the HLA-B*1502 allele), and it does not represent per se an absolute contraindication for the reintroduction of lamotrigine [75]. In recent years, the Food and Drug Administration (FDA) has released a warning to avoid lamotrigine in patients with known or suspected cardiac disorders because it appeared that lamotrigine could act as a class Ib antiarrhythmic [86]. However, these findings were based on in vitro data, and a task force was created to examine the existing literature regarding lamotrigine and possible cardiologic risks. A new advisory was released after a few months, removing the warning [87]. Lamotrigine appears to be a safe drug during pregnancy. The previously believed increased risk of palatal cleft or other major malformations in utero has not been confirmed in the latest research, making it a valuable maintenance treatment option for patients during pregnancy [88,89].

Lamotrigine’s therapeutic range is 3–15 μg/mL, and the level of recommendation for TDM is 2; it is essential to remember that valproate can increase the elimination half-life of lamotrigine, while carbamazepine can reduce it [5]. The importance of lamotrigine monitoring in the treatment of bipolar disorder remains unclear due to the scarcity of data; the existing literature shows that there might be a correlation between plasma concentrations and response (expressed with an improvement in depressive symptoms scales), but not all studies support these findings [90,91]. It is commonly accepted that blood count, urea, and electrolyte levels, as well as liver function, should be assessed before starting treatment, while lamotrigine plasma concentration may be checked in case of lack of response, suspected lack of adherence, or toxicity [5]. Some older studies also suggest a correlation between serum and saliva sampling in measuring lamotrigine concentrations, so this could be a good alternative in order to minimize the invasiveness of monitoring; however, it should be noted that these studies were conducted either in healthy volunteers or in patients with epilepsy, so the validity of saliva sampling in patients with psychiatric disorders is yet to be demonstrated [34,35,36]. Other researchers have recently proposed dried blood spots as an alternative to plasma sampling, with good results in terms of reliability and reduction of invasiveness [37].

Real-world research has shown that lamotrigine TDM, even when routinely followed, does not translate into dose adjustment by clinicians in case of samples under the recommended range [92]. Retrospective studies show that when used for the treatment of bipolar disorder, clinicians tend to keep daily lamotrigine dosages (and therefore plasma concentrations) at lower levels, which could result in underdosing or falling at the lower end of the therapeutic range, compared to epilepsy treatment [93,94].

## 3. TDM and Antipsychotics

Antipsychotics, also known as “neuroleptics”, are a diverse class of medications, primarily designed to manage symptoms associated with psychotic disorders. They are commonly prescribed for conditions such as schizophrenia, bipolar disorder, and other brain diseases characterized by hallucinations, delusions, and disorganized thinking. 

These drugs are usually divided into two main classes: typical (or first-generation antipsychotics, FGAs) and atypical (or second-generation antipsychotics, SGAs). While both types aim to modulate neurotransmitter activity, atypical antipsychotics are characterized by their reduced risk of extrapyramidal side effects such as parkinsonism and tardive dyskinesia and a better profile in terms of cognitive improvement [95]. On the other hand, treatment with some atypical antipsychotics has been associated with a substantial risk of metabolic effects, such as weight gain, hyperglycemia, and lipid dysregulation [96], as well as cerebrovascular adverse events, such as stroke [97], and cardiovascular adverse events [98]. 

In medical practice, haloperidol and phenothiazines are the most widely prescribed typical antipsychotics, and they act primarily as dopamine D2 receptor antagonists, managing the positive symptoms of psychosis. Conversely, clozapine, olanzapine, quetiapine, aripiprazole, and risperidone are the broadly used atypical antipsychotics and can treat both the positive and negative symptoms of psychosis. Antipsychotic prescription patterns can vary depending on the geographical region of the world, with a preference for older (and therefore less costly) molecules in developing countries, and on the use of the drug, with a preference for atypical antipsychotics in off-label settings [99,100,101,102].

The atypical antipsychotic mechanism of action goes beyond the D2 receptor blockade, involving serotonin (5-HT), muscarinic, adrenergic, and glutamatergic receptors. Besides the central role of dopamine in psychoses, serotonin (5-HT) and glutamate are strongly relevant for the physiopathology of these mental disorders. In 2018, a review revisited the mechanism of action of atypical antipsychotic drugs and, based on the different clinical characteristics of compounds belonging to the same category, grouped them into different levels of ‘atypia’. Indeed, a continuum spectrum of atypia has been proposed, ranging from risperidone, the least atypical (Level I), up to clozapine, the most atypical (Level III), while all others fall within these two extremes of the spectrum (Level II). The molecular targets increase moving from Level I to Level III, whereas clinical characteristics relate to their different molecular profiles. In detail, besides the canonical D2 and serotoninergic 5-HT2A/2C receptor antagonism, other mechanisms, such as D2 and 5-HT1 partial agonism, D3 antagonism, H1 antagonism, α2 antagonism, moderate muscarinic antagonism, M1 positive allosteric modulation, BDNF production, and GlyT blocking, have received particular attention to explain atypia [7,103].

TDM plays a crucial role in optimizing the effectiveness and safety of antipsychotic medications. It is worth noting that the plasma concentration of a drug is a good predictor for drug cerebral concentration, especially for lipophilic drugs, where the blood–brain barrier efflux transporters are poorly involved. By measuring the blood levels of these drugs, clinicians can ensure that patients receive the proper dosage, tailoring the treatment to individual needs. Furthermore, TDM helps identify variations in drug metabolism, potential interactions, and adherence issues, allowing for timely adjustments. This personalized approach enhances treatment outcomes while minimizing side effects and the risk of relapse in individuals with psychiatric disorders. It also helps simplifying therapeutic schemes and has the potential to reduce unnecessary polypharmacy [104].

PET studies have demonstrated that motor side effects, such as tremors and stiffness, may occur when more than 80% of the D2 receptors in the striatum are blocked [105]. Conversely, receptor occupancy between 65 and 80% seems to be the best condition for antipsychotic effectiveness, with a lower probability of inducing extrapyramidal side effects [106]. Notably, a correlation was found between D2 receptor occupancy and the plasma concentration of some antipsychotics [107], whereas such a relationship with dosage was less clear. This correlation between receptor occupancy and plasma concentrations was confirmed by different studies, which also showed that D2 receptor occupancy can be predicted by the antipsychotic concentration in plasma [108,109]. Studies have also found that the relationship between plasma concentration and D2 receptor occupancy is fit by a hyperbolic saturation curve, where risperidone and olanzapine, at higher concentrations, may exceed 80% of receptor occupancy. These curves show a good correlation between predicted and observed receptor occupancy and drug plasma concentration. The prediction of D2 receptor occupancy with plasma concentration is particularly valid for haloperidol and olanzapine, less so for risperidone, and not significant for clozapine [110]. For risperidone, the blood–brain barrier efflux transporters such as P-glycoprotein (P-gp) may be responsible for lowering its concentration in the brain, reducing the correlation, as mentioned earlier [111].

In vivo studies have recently analyzed the possible relationship between plasma concentration and receptor occupancy for other targets, such as the 5-HT2A receptor in the cortex and GlyT1 transporters. However, the information is too preliminary [112,113,114]. Furthermore, a statistically significant correlation between H1, muscarinic, and 5-HT2C receptor occupancies and metabolic side effects such as weight gain and diabetes mellitus type II has been demonstrated [115].

The dose–effect relationships of several drugs, including antipsychotics, vary considerably between patients, mainly owing to pharmacokinetic differences influenced by age, changes in the first-pass effect, and the induction or inhibition of the microsomal metabolic system. The primary source of pharmacokinetic variability is drug oxidation, a metabolic pathway catalyzed by the cytochrome P450 (CYP) enzyme system, whose activity varies widely among subjects because of environmental influences and genetic differences [116].

The AGNP group consensus guidelines [5] include haloperidol, amisulpride, clozapine, olanzapine, and some phenothiazines in level 1 recommendations regarding the routine monitoring of plasma concentrations. Many studies related to the variability between antipsychotic dose and plasma concentration have been carried out with clozapine, which nowadays is frequently monitored because of its relevant side effects. Predicting clozapine plasma concentration is challenging due to its inter-individual variability, contributed to by factors such as sex, age, weight, smoking, and concomitant use of other medications that influence CYP450 activity (e.g., CYP1A2) [117]. In particular, a fixed dose of clozapine of 400 mg/day showed substantial plasma concentration variability among patients [118]. Moreover, smoking lowers the plasma concentration of clozapine by inducing CYP1A2 [119], while CYP inhibitors, such as fluvoxamine, were shown to increase the plasma concentration of clozapine up to 10 times. On the other hand, co-administration with carbamazepine (a CYP3A4- and CYP1A2-inducing drug) resulted in a substantial decrease in the plasma concentration of clozapine [120]. 

Similar interactions were found with other atypical antipsychotics such as olanzapine and risperidone when they were co-administered either with carbamazepine or selective serotonin reuptake inhibitors fluoxetine and paroxetine, which are mostly CYP2D6- and CYP2C19-inhibiting drugs [121]. Regarding efficacy, the effective plasma clozapine window is still debated [122]. The study by Perry and colleagues showed for the first time that a clozapine plasma concentration greater than 350 ng/mL in treatment-resistant patients with schizophrenia resulted in a 64% clinical response, while below this level, the response was only 22%. Other studies have also confirmed a cut-off point for clozapine efficacy at 350 ng/mL [123] or 420 ng/mL [124]. According to the AGNP-TDM expert group consensus guidelines [5], the recommended therapeutic range of clozapine plasma concentration is 350–600 ng/mL. Plasma concentrations greater than 1000 ng/mL can increase the risk of epileptic seizures. Therapeutic drug monitoring is also strongly recommended in pediatric patients under clozapine treatment [125]. Furthermore, dose adjustment in female individuals might also be reasonable, according to sex-related differences in serum concentrations [126].

Regarding olanzapine, studies have investigated the relationship between the daily olanzapine dose and plasma concentrations, showing that the latter increases linearly with the daily oral dose [127,128]. Moreover, some authors have demonstrated a linear relationship between the prescribed daily dose and the plasma concentration of the primary N-desmethyl olanzapine metabolite [129]. At commonly used daily olanzapine doses (5–30 mg/day), mean plasma concentrations range from 10 to 54 ng/mL. Considerable inter-patient variability has been observed, depending on co-medications, inter-individual variability in drug metabolism and/or clearance, and gender [130].

The therapeutical range of plasma concentration for amisulpride has been poorly investigated. A study by Piacentino [131] considered optimal therapeutic plasma amisulpride concentrations of about 367 ng/mL to be associated with stable clinical improvement [131]. However, further investigations are required to verify the association between plasma concentrations and responses and whether there is a correlation between plasma drug concentrations and prolactin levels. This information would help support the therapeutic range of 100–320 ng/mL proposed by the AGNP-TDM.

These data clearly show that regular TDM assessments contribute to the precision and success of antipsychotic therapy, fostering a balance between therapeutic benefits and potential adverse effects.

In regard to “third generation” antipsychotics (TGAs), and in particular aripiprazole (level 2 of recommendation), the established recommended therapeutic range is 120–270 ng/mL (180–380 ng/mL for its active metabolite dehydroaripiprazole), even though the relationship between concentration and efficacy, as well as the correlation between concentration and adverse effects, remains unclear [132]. However, a recent study by Tien and colleagues, based on a Chinese population of patients taking aripiprazole, found a higher response rate in patients with a serum concentration over 300 ng/mL, suggesting that increasing aripiprazole concentrations above the current recommended range could potentially improve patients’ response to treatment [133].

In regard to alternatives to blood sampling, in the past decade, there has been an increased interest in evaluating less-invasive methods for antipsychotics TDM. Dziurkowska and Wesolowski implemented a novel method to enable the quantification of olanzapine, risperidone, clozapine, quetiapine, and aripiprazole in small biological samples such as saliva with good accuracy, although the number of patients included in this study was relatively small [134]. Another group evaluated the stability of oral fluid samples of chlorpromazine, levomepromazine, cyamemazine, clozapine, haloperidol, and quetiapine and found that under certain conditions (i.e., at a stable 4 °C temperature, in a dark environment, and with low acidic concentrations), the samples exhibited good stability over time, with ranges between 14 and 146 days [135]. Saliva sampling has also been recently proposed for amisulpride [136]. Urine sampling has been evaluated as a monitoring technique for risperidone, haloperidol, quetiapine, and olanzapine [137]. Minimally invasive sampling methods, such as dried blood spots sampling, have also been evaluated in recent years for several antipsychotic medications [138]. Table 2 summarizes the main sampling methods of some of the most used antipsychotic medications, along with the therapeutic reference ranges and the AGNP recommendation levels. A recent preclinical study by Yan and colleagues implemented a smart lollipop-like sensing system that can be connected to a smartphone and that could decentralize TDM for clozapine samples, potentially allowing patients to monitor themselves at home [139]. The further development of these methods could represent a cost-effective and non-invasive way to strengthen the relationship between clinicians and patients, therefore leading to a more personalized and human-centered approach.

## 4. TDM and Antidepressant Medications

TDM for antidepressant medications can improve treatment optimization in order to monitor treatment adherence and avoid or limit ADRs or toxicity; however, the use of TDM for this wildly used class of drugs is limited compared to other drugs, such as mood stabilizers or antipsychotic medications [140]. TDM currently applies to numerous antidepressants, and serum drug concentrations might represent a better index than drug dosage [141].

For Tricyclic Antidepressants (TCAs), TDM’s usefulness has been wildly accepted due to the risk of several troublesome side effects and for safety reasons, mainly because they are potentially cardiotoxic. Most of them fall into category 1 of TDM recommendation, except for trimipramine and desipramine, which are included in category 2 [5]. An older study by Müller and colleagues on the use of TDM in patients taking TCAs showed that despite clinicians often being “non-compliant” to TDM recommendations, there was a clinical benefit in performing early TDM in patients, with an increase in depressive symptoms scales, although a direct effect on cost-effectiveness was not found [142]. A focus on TDM in antidepressants confirmed the utility of TDM in TCAs, mainly as an aid for the clinician to avoid intoxications that may be deadly for patients [143].

Regarding selective serotonin reuptake inhibitors (SSRIs), there is a correlation between serotonin reuptake inhibition and the concentration of the drug in plasma [144]. However, most serotonin reuptake inhibition occurs at what is usually considered the “minimum effective dose” [141]. SSRIs fall either in category 2 (escitalopram, paroxetine, fluvoxamine, sertraline) or 3 (fluoxetine) of evidence regarding TDM recommendations. The only SSRI for which there is a level 1 recommendation is citalopram, which has a therapeutic range between 50 and 110 ng/mL [5]. For this drug, a reduction in hospitalization (and a subsequent cost reduction) when using TDM in the early stages of treatment was observed, and a positive correlation between plasma concentrations and response to treatment has been shown [145,146]. For other SSRIs, TDM might have a role in checking for treatment adherence or fast and slow metabolizers. It could also be helpful in assessing the presence of its metabolite, norfluoxetine, after fluoxetine discontinuation [141].

Serotonin–norepinephrine reuptake inhibitors (SNRIs), specifically, venlafaxine and duloxetine, are included in the category 2 level of recommendation [5]. TDM might be helpful for dose titration in both venlafaxine and duloxetine treatment, especially in the elderly [147,148]. Other antidepressant medications have not been thoroughly studied regarding TDM’s usefulness. Mirtazapine exhibits a linear (but weak) relationship between plasma concentration and oral doses; however, a clear concentration–effect relationship has not yet been established [149]. Bupropion TDM may help increase treatment safety and effectiveness, but its monitoring is not routinely performed; measuring plasma concentrations of monoamine oxidase inhibitors (MAOIs) is costly and rarely feasible [141]. The newer antidepressant vortioxetine has been classified in the category 2 level of recommendation; however, the literature on the utility of TDM for this novel medication is still lacking [150].

The development of alternatives to blood samples has also been carried out for antidepressant medications. The use of the Supported Liquid Extraction method has proven to be good in measuring saliva concentrations of amitriptyline, mianserin, duloxetine, mirtazapine, sertraline, citalopram, and venlafaxine [151]. Dried-saliva spot sampling has been proposed as a valid method for measuring the concentrations of fluoxetine, venlafaxine, O-desmethylvenlafaxine, citalopram, sertraline, and paroxetine, representing a valid alternative to blood drawing [152]. Oral fluid microsampling also gave satisfying results in the monitoring of sertraline, fluoxetine, citalopram, and vortioxetine [153]. Urine can be used as a matrix for monitoring escitalopram, citalopram, fluoxetine, paroxetine, and bupropion, and it has also been proposed for the monitorization of fluvoxamine and moclobemide [154,155,156,157,158,159,160,161]. For TCAs, urine sampling can be used, even though mostly for forensic studies rather than for routinary monitoring [162]. For vortioxetine, both urine and saliva could be used with reliability for monitoring [163]. As for in silico monitoring, the algorithm XGBoost has shown promising results in predicting the best medication regimen in patients with depression [164]. Further research is needed to confirm these findings, in order to achieve a wider implementation of these methods in clinical practice. A summary of the main sampling methods used for the most used antidepressants can be found in Table 3.

## 5. Conclusions

TDM is a valuable asset in the correct treatment of patients with psychiatric disorders, as it helps to choose the appropriate drug dosages to maximize the desired effects and minimize ADRs, while checking for correct adherence to the treatment. In our review, we analyzed the role of TDM in the main pharmacological classes of psychiatric medications and how it should be implemented in clinical practice. We did not include the use of TDM for anxiolytic medications, for drugs such as methylphenidate or atomoxetine, or for drugs used to treat substance use disorder because the usefulness of monitoring these particular drugs is still uncertain, as these medications have level 3 or 4 recommendations in the AGNP-TDM consensus guidelines [150]. Therefore, more research is needed to study the utility and the possibility of implementing TDM in these drug categories. Future research should also focus on implementing cost-effective and less-invasive methods to perform TDM, such as saliva sampling, to increase patients’ adherence to treatment and monitoring.

## Figures and Tables

**Table 1:** Mood stabilizers used in psychiatric disorders with reference ranges, AGNP recommendation levels, and their main sampling methods.

Drug	Class	Therapeutic Drug Range (Blood)	TDM AGNP Recommendation Levels	Preferred Sampling Method	Other Sampling Methods
Lithium	Mood stabilizer	0.5–1.2 mmol/L	1	Plasma	Saliva, urine, sweat, interstitial fluid, dried blood/plasma spots [23,24]
Valproate	Antiseizure medication/mood stabilizer/migraine prevention	50–100 μg/mL	2	Plasma	Saliva, urine, dried blood spots [25,26,27]
Carbamazepine	Antiseizure medication/mood stabilizer/neuropathic pain	4–12 μg/mL	1	Serum	Saliva, urine [28,29,30,31,32]
Oxcarbazepine	Antiseizure medication/mood stabilizer	10–35 μg/mL	2	Plasma	Urine [33]
Lamotrigine	Antiseizure medication/mood stabilizer	3–15 μg/mL	2	Plasma	Saliva, dried blood spots [34,35,36,37]

**Table 2:** Frequently used antipsychotic medications, with their therapeutic reference ranges, AGNP recommendation level, and sampling methods, including experimental ones. FGA: first generation antipsychotics; SGA: second generation antipsychotics; TGA: third generation antipsychotics.

Drug	Class	Therapeutic Drug Range (Blood)	TDM AGNP Recommendation Levels	Preferred Sampling Method	Other Sampling Methods
Amisulpride	SGA	100–320 ng/mL	1	Plasma	Saliva, dried blood spots [136,138]
Aripiprazole	TGA	100–350 ng/mL	2	Plasma	Saliva, dried blood spots [134,138]
Chlorpromazine	FGA	30–300 ng/mL	2	Plasma	Saliva [135]
Clozapine	SGA	350–600 ng/mL	1	Plasma	Saliva, dried blood spots [134,135,136,138]
Haloperidol	FGA	1–10 ng/mL	1	Plasma	Saliva, dried blood spots, urine [135,137,138]
Olanzapine	SGA	20–80 ng/mL	1	Plasma	Saliva, dried blood spots, urine [134,135,137,138]
Quetiapine	SGA	100–500 ng/mL	2	Plasma	Saliva, dried blood spots, urine [134,135,137]
Risperidone	SGA	20–60 ng/mL	2	Plasma	Saliva, dried blood spots, urine [134,137,138]

**Table 3:** Some of the main antidepressant medications with their therapeutic reference range, the AGNP recommendation level, and the most used and the experimental sampling methods. SSRI: selective serotonin reuptake inhibitor; SNRI: serotonin–norepinephrine reuptake inhibitor; NDRI: norepinephrine–dopamine reuptake inhibitor; TCA: tricyclic antidepressant; NaSSA: noradrenergic and specific serotonergic antidepressants; MAOI: monoamine oxidase inhibitor; SMS: serotonin modulator and stimulator.

Drug	Class	Therapeutic Drug Range (Blood)	TDM AGNP Recommendation Levels	Preferred Sampling Method	Other Sampling Methods
Amitriptyline	TCA	80–200 ng/mL	1	Plasma	Saliva, urine [162]
Bupropion	NDRI	10–100 ng/mL	2	Plasma	Urine [156]
Citalopram	SSRI	50–110 ng/mL	1	Plasma, serum	Saliva, dried saliva spots, urine [151,152,153,159]
Clomipramine	TCA	230–450 ng/mL	1	Plasma	Urine [162]
Desipramine	TCA	100–300 ng/mL	2	Plasma	Urine [162]
Duloxetine	SNRI	30–120 ng/mL	2	Plasma	Saliva [151,152]
Escitalopram	SSRI	15–80 ng/mL	2	Plasma, serum	Urine [154,155]
Fluoxetine	SSRI	120–500 ng/mL	3	Plasma, serum	Urine, dried saliva spots [152,153,159]
Fluvoxamine	SSRI	60–230 ng/mL	2	Plasma, serum	Urine [157,161]
Imipramine	TCA	175–300 ng/mL	1	Plasma	Urine [162]
Mirtazapine	NaSSA	30–80 ng/mL	2	Plasma	Saliva [151]
Moclobemide	MAOI	300–1000 ng/mL	3	Plasma	Urine [158]
Nortriptyline	TCA	70–170 ng/mL	1	Plasma	Urine [162]
Paroxetine	SSRI	20–65 ng/mL	3	Plasma, serum	Urine, dried saliva spots [152,160]
Sertraline	SSRI	10–150 ng/mL	2	Plasma, serum	Saliva, dried saliva spots [151,152,153]
Venlafaxine	SNRI	100–400 ng/mL	2	Plasma	Saliva, dried saliva spots [151,152]
Vortioxetine	SMS/SSRI	15–60 ng/mL	2	Plasma	Saliva, urine [153,163]
